# Editorial: Adrenal insufficiency: diagnostic approaches, treatments, and outcomes, volume II

**DOI:** 10.3389/fendo.2025.1629881

**Published:** 2025-06-13

**Authors:** Agnieszka Pazderska, Carl D. Malchoff, Elena Valassi

**Affiliations:** ^1^ Department of Endocrinology, St. James’s Hospital, Dublin, Ireland; ^2^ School of Medicine, Trinity College Dublin, Dublin, Ireland; ^3^ Department of Medicine, University of Connecticut Health Center, Farmington, CT, United States; ^4^ Endocrinology and Nutrition Department, Germans Trias i Pujol Hospital and Research Institute, Badalona, Spain; ^5^ Centro de Investigación Biomédica en Red de Enfermedades Raras (CIBERER), Universitat Internacional de Catalunia (UIC), Barcelona, Spain

**Keywords:** adrenal insufficency, corticosteriod, hydrocortisone (HC), adrenal crisis, CIRCI (critical illness related corticosteroid insufficiency)

Adrenal insufficiency (AI) was first described by Thomas Addison in 1855; it was an invariably fatal condition at the time (1). Despite many advances in recent years, several aspects of the diagnosis and treatment of AI remain challenging. The diagnosis of the disease is often delayed by months resulting in many patients presenting with acute adrenal crisis. Modern cortisol replacement regimes fail to mimic the physiological circadian rhythm of cortisol secretion and currently there are no curative options for primary adrenal insufficiency (PAI). This Research Topic aims to highlight some of the diagnostic and management challenges in adrenal insufficiency and present an overview of the current knowledge and future directions.

In the developed world, autoimmune adrenalitis (PAI) is the most common culprit, with iatrogenic causes also on the rise. In this Research Topic, we present case reports of AI due to use of various medications. Choo et al. report a patient presenting with severe, symptomatic hypercalcaemia due to adrenal insufficiency precipitated by fluconazole and previous exogenous glucocorticoid use. Wu et al. describe two patients presenting with severe symptomatic hypokalemia caused by the use of electronic cigarettes containing etomidate. Both patients presented with hypertension, hypokalemia, low cortisol, high ACTH, low renin, low aldosterone and bilateral adrenal gland thickening on abdominal imaging. Etomidate is known to reduce the synthesis of cortisol, corticosterone and aldosterone by blocking 11β-hydroxylase (CYP11B1). Accumulation of 11-deoxycorticosterone-which has mineralocorticoid activity- can explain the clinical presentation.

Despite advances in glucocorticoid replacement, adrenal crisis remains an issue in patients with AI (2). Patient education and adequate self-management are important aspects in the prevention of adrenal crises. Llahana et al. aim to develop a theory-informed, digital behaviour change intervention (DBCI) to support self-management in patients with AI to complement usual care for such patients. The authors describe the systematic process they will follow to address determinants of self-management behaviours in patients with AI and subsequently selecting salient behaviour change techniques to use when developing a tailored digital intervention.

In practice, conventional therapy with hydrocortisone or cortisone acetate leads to supraphysiological cortisol peaks which in longer term culminates in complications (3). In an attempt to maintain more physiological cortisol concentrations, a dual release hydrocortisone (DR-HC) preparation, Plenadren^®^, has been developed. In an observational study, Bioletto et al. attempts to assess the impact of DR-HC on skeletal health by comparing bone-related parameters in patients with PAI treated with conventional therapy (C-GC) (n=13) and DR-HC (n=14) at an equivalent daily dose. Bone turnover markers did not differ significantly between the groups. However, patients treated with DR-HC had higher bone mineral density at lumbar spine and femoral neck, as well as trabecular bone scores, compared to those on C-GC. 3 patients in the C-GC group had vertebral fractures (total of 9 fractures) versus none in the DR-HC group. Overall, the results may suggest a better bone safety profile of DR-HC compared to C-GC.

Wolff et al. discus the genetic underpinning of PAI, including monogenic forms of the disease, as well as discussing the role of genetic polymorphism in the aetiology of autoimmune PAI. The world’s first Genome Wide Association Study on autoimmune PAI identified nine genetic regions that were predicted to explain 40% of the genetic susceptibility for autoimmune PAI. Furthermore, the authors discuss how the knowledge of the genetic basis for PAI can be used in the future in predicting the disease susceptibility in high-risk individuals and in helping to identify subjects who may have monogenic forms of the disease. The advancement of the knowledge on how to identify at-risk individuals, paired with the understanding of the autoimmune processes involved in the pathophysiology of the disease may help create targeted interventions designed to prevent PAI development.

The most common aetiology of AI is iatrogenic, with exogenous steroid use being a major contributor (4). However, with the recent rise in opioid use, the inhibitory effects of this class of medications on the hypothalamic-pituitary-adrenal (HPA) axis is increasingly encountered. Patel and Ben-Shlomo discuss diagnostic and management considerations in opioid-induced AI. The authors suggest that all physicians treating patients with chronic opioids should evaluate them periodically for symptoms of adrenal insufficiency. Since many patients who receive chronic opioids can not stop them due to severe pain, screening with AM cortisol is less informative. The authors suggest diagnosis should be confirmed with the high dose (250 µg) ACTH^1–24^ stimulation test, after avoiding administration of opioids for several hours prior to the testing, if possible. Cessation or reduction of opioid doses can lead to recovery of HPA axis; however, the opioid dose at which this occurs remains unknown.

The COVID-19 pandemic which started in December 2019 has seen more than 778 million confirmed cases worldwide (5). In a cross-sectional study, Porntharukchareon et al. evaluated the prevalence of hypocortisolism, diagnosed using low dose (1µg) ACTH^1–24^ stimulation test, three months after radiologically confirmed COVID-19 pneumonia. Of the 41 patients evaluated, eleven (27%) had hypocortisolism. Only five of the eleven patients received dexamethasone for treatment of the acute pneumonia. Increased BMI was identified as a risk factor for hypocortisolism.

Critical Illness-Related Corticosteroid Insufficiency (CIRCI) is a condition describing impairment of the HPA axis developed following critical illness (6). Sobolewska et al. discuss the pathophysiology, diagnostic pitfalls and individualized management of this condition. The current recommendation is to use total serum cortisol measurement <10 µg/dL (276 nmol/L) or change in baseline cortisol of less than 9 µg/dl (248 nmol/l) at 60 min after 250µg ACTH^1–24^ administration, to diagnose CIRCI. However, decrease in cortisol plasma binding protein (CBG) and its binding affinity, often proportional to the severity of the illness, increases the distribution volume for cortisol, thus reducing the incremental response in total plasma cortisol to ACTH injection. This is highly predictive of mortality, but should not be used to identify patients who should be treated with exogenous glucocorticoids. The authors review findings from three major randomised controlled trials (ADRENAL, APROCCHSS and CORTICUS) on corticosteroid use in patients with septic shock and their effect on mortality.

Patients with primary hyperaldosteronism (PA) have increased risk of chronic kidney disease (7). Adrenalectomy is a treatment option for those with unilateral source of aldosterone excess. A significant proportion of patients have a significant eGFR decline after the surgery which is at least partly related to reduction in the hyperfiltration and unmasking of CKD after aldosterone excess has been corrected. Ma et al. report clinical outcomes and changes in renal function after adrenalectomy in patients with PA across different age groups (<40, 40-60, >60 years old). Patients aged <40 years had the highest rate of complete clinical success but the complete biochemical success was similar between the age groups. eGFR declined similarly in all three age groups in the short- and long-term. Preoperative systolic blood pressure, plasma aldosterone concentration and hypertension duration were significant predictors of postoperative renal function impairment.

Fertility and parity in women with PAI is reduced (8). O’Murchadha et al. discuss the current understanding of factors that may affect fertility and parity in women with autoimmune PAI, including premature ovarian insufficiency, co-existence of other autoimmune conditions, the potential role of the impaired adrenal sex-steroid production, and psychosocial factors and libido. The authors summarize studies reporting pregnancy outcomes in women with AI showing higher rates of caesarean section and somewhat increased risk of premature birth in this population.

We trust that this Research Topic provides valuable clinical and scientific updates to inform clinicians on important aspects of managing patients with adrenal insufficiency.
